# 84539 Developing a Multilevel Intervention to Increase Hepatitis C Virus Screening of Baby Boomers in Primary Care

**DOI:** 10.1017/cts.2021.551

**Published:** 2021-03-30

**Authors:** Monica Kasting, Susan Rawl

**Affiliations:** 1 Purdue University; 2 Indiana University School of Nursing

## Abstract

ABSTRACT IMPACT: This research will improve human health by increasing screening for hepatitis C virus, thereby decreasing morbidity and mortality from hepatitis C-related disease. OBJECTIVES/GOALS: The worldwide incidence of liver cancer increased 75% from 1990 to 2015 due, in part, to chronic hepatitis C virus (HCV) infection. Individuals born 1945-1965 (baby boomers) have five times the prevalence of HCV infection compared to other birth cohorts, but fewer than 15% of this cohort have ever been screened. METHODS/STUDY POPULATION: Effective interventions to increase HCV screening among baby boomers are urgently needed. In partnership with a provider advisory board and a community advisory board, we will develop a multilevel intervention designed to increase HCV screening that will be delivered to both providers and patients in primary care. We will assess whether the intervention is feasible, acceptable, and usable from the perspectives of the target audiences (providers and patients) by conducting Concurrent Think Aloud (CTA) interviews with eight patients and eight providers. RESULTS/ANTICIPATED RESULTS: While the specific content of both intervention components will not be finalized until the completion of the study, we envision that the provider-level intervention will likely include a one-time educational session and monthly performance feedback provided via e-mail reporting each provider’s HCV screening rates. The patient-level intervention may include mailed reminder letters prior to a scheduled clinic visit informing them that HCV screening is recommended and a tablet-based in-clinic computer program to educate, engage, and activate patients to be screened. DISCUSSION/SIGNIFICANCE OF FINDINGS: The goals of this project are to: 1) develop an acceptable, feasible, and usable multilevel intervention aimed at increasing HCV screening in primary care; and 2) understand the relationship between the intervention components and HCV screening; and 3) reduce HCV-related morbidity and mortality.

